# Blastoderm segmentation in *Oncopeltus fasciatus* and the evolution of insect segmentation mechanisms

**DOI:** 10.1098/rspb.2016.1745

**Published:** 2016-10-12

**Authors:** Reut Stahi, Ariel D. Chipman

**Affiliations:** The Department of Ecology, Evolution and Behavior, The Alexander Silberman Institute of Life Sciences, The Hebrew University of Jerusalem, Edmond J. Safra Campus, Givat Ram 91904, Jerusalem, Israel

**Keywords:** segmentation, transcription factors, evo-devo, insects, blastoderm

## Abstract

Segments are formed simultaneously in the blastoderm of the fly *Drosophila melanogaster* through a hierarchical cascade of interacting transcription factors. Conversely, in many insects and in all non-insect arthropods most segments are formed sequentially from the posterior. We have looked at segmentation in the milkweed bug *Oncopeltus fasciatus.* Posterior segments are formed sequentially, through what is probably the ancestral arthropod mechanism. Formation of anterior segments bears many similarities to the *Drosophila* segmentation mode. These segments appear nearly simultaneously in the blastoderm, via a segmentation cascade that involves orthologues of *Drosophila* gap genes working through a functionally similar mechanism. We suggest that simultaneous blastoderm segmentation evolved at or close to the origin of holometabolous insects, and formed the basis for the evolution of the segmentation mode seen in *Drosophila*. We discuss the changes in segmentation mechanisms throughout insect evolution, and suggest that the appearance of simultaneous segmentation as a novel feature of holometabolous insects may have contributed to the phenomenal success of this group.

## 1. Background

Insects are the most diverse taxon on the Earth and are characterized by a highly conserved body plan [1]. Despite myriad variations in lifestyle, ecology and feeding, all insects have a segmented body divided into three distinct body regions (tagmata): a head composed of three pre-oral and three gnathal segments, a thorax composed of three leg-bearing segments, two of which usually also bear wings, and an abdomen with 9–11 segments. This conservation of general body plan masks a diversity of mechanisms employed to establish it during embryogenesis [2]. These diverse mechanisms use a fairly well-conserved suite of developmentally relevant genes acting in different cellular contexts.

Of these differences in developmental mechanisms, probably the best-known contrast is that between long and short germ insects [2–5]. The terms describe what extent of the segmented germ-band is determined prior to gastrulation: long, intermediate or short germ, corresponding to all, some or almost none of the segments patterned prior to gastrulation [3]. The differences in germ type have significant implications for many aspects of embryonic development, and specifically for the mode of segmentation. The mode of segmentation that has become almost paradigmatic for insects is the long germ development mode of *Drosophila melanogaster* [6–8]. In this extreme long germ mode, all 15 embryonic segments are determined almost simultaneously via a series of interacting transcription factors (the so-called maternal, gap, pair-rule and segment-polarity genes) that pattern the entire extent of the germ-band prior to gastrulation [6,7]. By contrast, in short or intermediate germ development, most segments are added sequentially after gastrulation, usually from a posterior growth zone. Many non-insect arthropods use the Delta-Notch signalling pathway as the main pathway that generates a temporally repeated pattern, which is then translated into a spatially repeated pattern that sequentially creates segments from a growth zone [9–11]. In hemimetabolous insects, Delta-Notch signalling is also apparently involved in segmentation [12–14], although it is not clear to what extent.

This distinction is somewhat of an over-simplification, and the different modes are not as discrete as is sometimes thought. Long germ development is often equated with simultaneous segmentation (as in *Drosophila*), whereas short germ development is often equated with sequential segmentation. A more nuanced analysis shows that in fact, many insects use two mechanisms for segment generation. The anterior-most segments (normally the head and all or some of the thorax) appear during the blastoderm stage, while the remaining segments (normally constituting at least the abdomen) appear sequentially from a growth zone during the germ-band stage.

**Figure 1.**
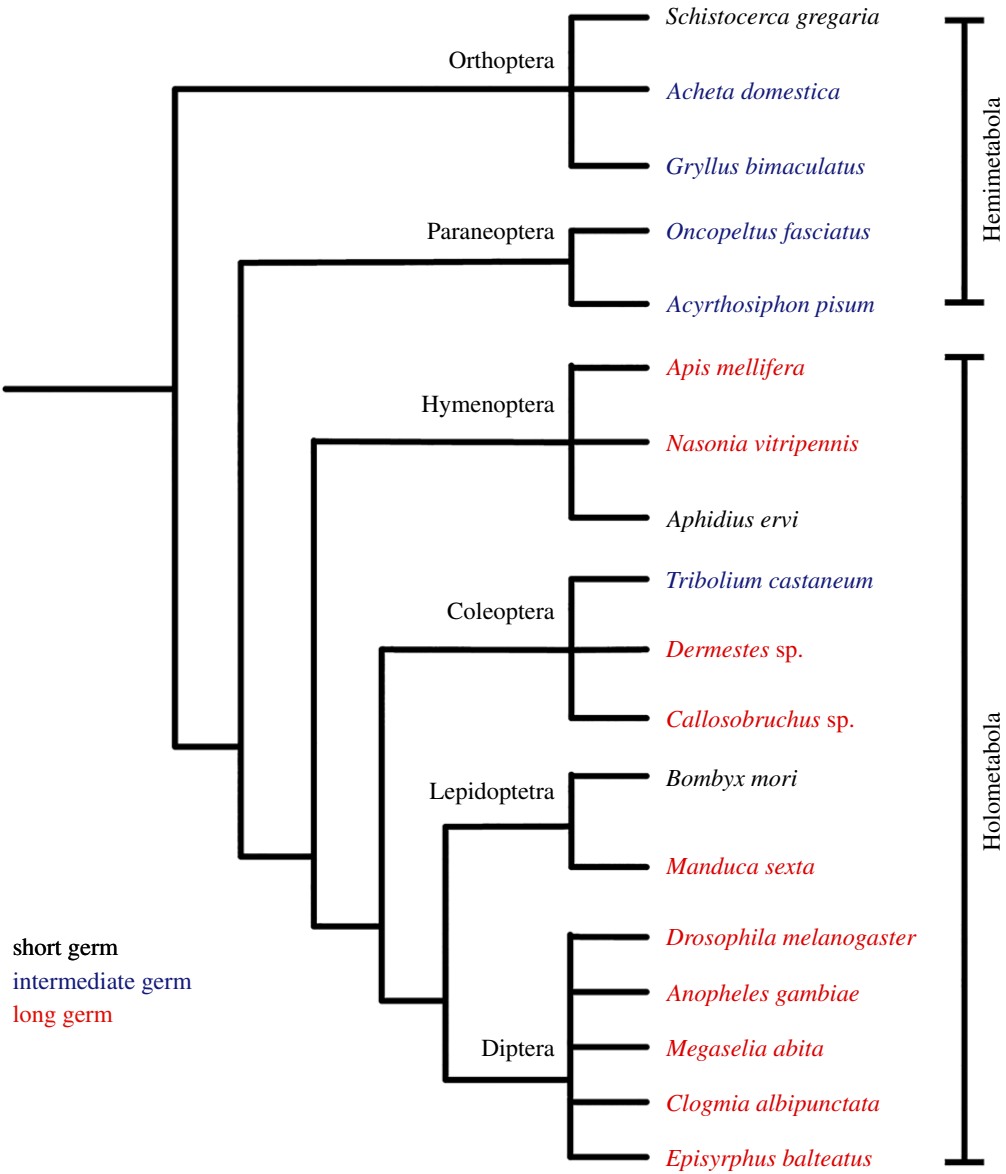
Phylogenetic spread of germ types in selected insects. Redrawn and modified from Ten Tusscher [15].

The distribution of long germ patterning among different insect groups makes it difficult to reconstruct its evolutionary history unequivocally (figure 1). All members of the early branching hemimetabolous insects (those insects with direct development through a series of larval instars) develop through short or intermediate germ development, with sequential segmentation in the posterior segments, as do virtually all non-insect arthropods. Thus, it seems very likely that the ancestral mode for arthropods is short or intermediate germ development [2,16]. Within the more recently evolved holometabolous insects (those with indirect development that includes a pupal stage and dramatic metamorphosis), long germ development is found in all four major orders, but not in all species within these orders [5]. This leaves open two formal possibilities; either long germ development has evolved several times within holometabolous insects or long germ development appeared at (or close to) the origin of the holometabolous insects and has been secondarily lost several times. Discriminating between these two possibilities is crucial for understanding the evolution of segmentation in insects, and for understanding how the conserved set of developmental genes has been redeployed at key transitions in insect evolution [2].

In order to understand how long germ development has evolved from short germ, we need to look for a species that is close enough to Holometabola and displays both sequential and simultaneous segmentation. A model organism that fits these two requirements is the milkweed bug *Oncopeltus fasciatus*. Hemiptera, to which *Oncopeltus* belongs, is the closest hemimetabolous order to Holometabola [17]. The embryogenesis of *Oncopeltus* [18,19] follows two main stages: an earlier blastoderm stage, which superficially resembles that of *Drosophila*, and a later germ-band stage, which is characteristic of sequentially segmenting insects. During the blastoderm stage, the anterior of the embryo is patterned down to the segment-polarity gene level, and six segments, corresponding to the gnathal and thoracic segments, are formed simultaneously. The anterior head segments are probably formed using a different, more ancient mechanism [20–22].

In this work, we have analysed the blastoderm segmentation process in *Oncopeltus* in order to understand the transition from short to long germ segmentation. We have chosen to focus on a sample of genes that have been previously studied in *Drosophila* and other arthropods, as well as in previous work on *Oncopeltus*. The pair-rule gene *even-skipped* (*eve*) is expressed in an early broad domain in *Drosophila* and resolves to a pair-rule periodicity through clearing of intersegmental stripes. In other arthropods, it has a key early role in generating a repeated pattern. Delta is a ligand of the notch pathway, with no known role in segmentation in *Drosophila* but a suggested role in other arthropods (see above). The segment-polarity genes *engrailed/invected* and *wingless* are expressed in segmental stripes in *Drosophila* and in virtually all other arthropods where they have been examined. In analysing the involvement of these genes in *Oncopletus* blastoderm segmentation, we show that it bears significant similarities to the blastoderm segmentation process in *Drosophila,* suggesting that simultaneous segmentation in the blastoderm evolved before the holometabolous radiation.

## 2. Material and methods

Animal husbandry, embryo collection and fixation, RNAi and *in situ* hybridization were all performed as previously described [23], except that embryo collections were mostly in ½ h windows instead of 2 h windows. All genes used have been previously published. GenBank accession numbers for the relevant genes are: *Delta*: KU870474; *caudal*: KU870475; *even-skipped*: AY870400; *invected*: AY460340; *giant*: GU123166; *Krüppel*: AY627357; *hunchback*: AY460341.

## 3. Results

### (a) Expression patterns of the segmentation genes

**Figure 2.**
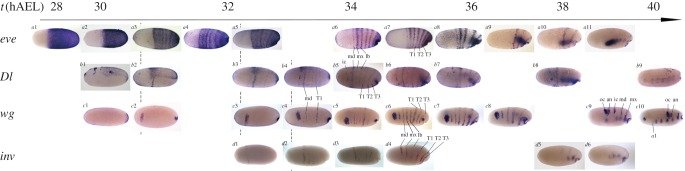
The development of the expression pattern of four segmentally expressed genes: (i) *even-skipped*, (ii) *Delta*, (iii) *wingless* and (iv) *invected.* The time axis (in hours after egg lay) is approximate owing to small variability among clutches. Embryos in the same column are of the same age. In all cases, anterior is to the left and dorsal to the top except for *b*9, which is a dorso-lateral view. Dashed lines connect embryos from the same clutch. The identity of the segments is marked on representative embryos: A1, first abdominal segment; an, antennal; ic, intercalary; lb, labial; md, mandibular; mx, maxillary; oc, ocular; T1–T3, first to third thoracic segment.

We looked at four genes expressed during the process of blastodermal segmentation: *invected* (*inv*), *wingless* (*wg*), *even-skipped* (*eve*) and *Delta* (*Dl*). For each gene, we examined the expression pattern over time at closely spaced intervals between 30 and 40 h after egg laying (hAEL) at 25°C (figure 2). We also examined their mutual interactions and the effects of knocking down gap genes on their expression pattern.

In order to synchronize the expression patterns of the four segmentation genes, we carried out some of the analyses on single clutches, which were separated into four groups, each stained for one of the segmentation genes. In other cases, we used the extent of germ-band invagination as a proxy for developmental age. Preliminary analyses showed that although there is notable variability in developmental age among clutches of the same age (in hAEL), variability within a single clutch at these stages is negligible in comparison.

#### (i) even-skipped

The expression of *even-skipped* (*eve*) has been previously reported by Liu & Kaufman [24]. Our results are mostly consistent with their report, but we dispute some of the details. Uniform expression of *eve* in the posterior 2/3 of the embryo begins before 30 hAEL (figure 2*a*1), and contracts to the posterior half by 30 hAEL (figure 2*a*2). From that stage and up to 34 hAEL, *eve* expression begins a clearing process ending with six distinct segmental stripes (figure 2*a*3–7). Contrary to Liu and Kaufman's claim that the clearing process occurs sequentially, from the anterior to the posterior, figure 2 shows that the clearing does not occur in a strict A–P sequence. The first segments that undergo clearing are the first two thoracic segments, followed by the labial segment. The maxillary and mandibular segments separate and sharpen next, with the maxillary stripe being significantly stronger and broader. Finally, the third thoracic segment separates from the remaining posterior expression domain, which will become the growth zone. Blastoderm invagination starts shortly afterwards and segmental expression of *eve* is maintained until the end of the process.

#### (ii) Delta

Expression of *Delta* (*Dl*) appears at around 30 hAEL as a dorsal-posterior patch (figure 2*b*1). Shortly afterwards, additional expression appears in a clear ring that surrounds the whole embryo, and corresponds to the future mandibular segment (figure 2*b*2). At 33–34 hAEL, five additional weak stripes appear posterior to the mandibular ring (figure 2*b*3–4). By 35 hAEL, all six post-mandibular segmental stripes are clearly expressed with an additional anterior lateral expression domain added, probably corresponding to the intercalary segment (figure 2*b*5–6). As invagination begins expression fades. Segmental expression of *Dl* can no longer be seen in the gnathal and thoracic segments during the germ-band stage. However, pro-neural expression of *Dl* appears even before the end of invagination, as a series of spots in the blastoderm, and persists throughout the germ-band stage (figure 2*b*9).

#### (iii) wingless

The expression of *wingless* (*wg*) begins at 30 hAEL as a posterior cap (figure 2*c*1). By 32 hAEL bilateral elliptical expression patches appear on the sides of the embryo, and posterior cap resolves to a small domain in the future invagination site (figure 2*c*2–3). Between 33 and 36 hAEL, seven more stripes of expression appear gradually in non-sequential order (figure 2*c*4–7). The first segmental stripes to appear are the mandibular and first thoracic segment at approximately 34 hAEL, followed at approximately 35 hAEL by the intercalary, maxillary, labial and second thoracic segment, and finally, at approximately 35 hAEL, the third thoracic stripe appears leading to nine distinct expression domains, including a posterior expression area that marks the invagination site and the location of the future growth zone.

Tracing the expression of the anterior elliptical patch during invagination (figure 2*c*9–10) uncovers a separation of the patch into two distinct domains that we interpret as the ocular and antennal segments. We note also that segmentation from the growth zone begins before the end of invagination, so that a tenth segment—the first abdominal segment—appears during that phase (figure 2*c*10).

#### (iv) invected

The gene now identified as *invected* (*inv*) was previously identified as *engrailed*. It is a paralogue of *engrailed* [25] and has an apparently similar role. It is the last gene to appear segmentally in the segmentation cascade. The first segmental stripe appears at 33 hAEL on the dorsal side of the embryo (figure 2*d*1); that first stripe will give rise to the mandibular segment. Between 35 and 37 hAEL, the remaining stripes appear gradually—maxillary first, followed by the first two thoracic segments and finally, the labial and third thoracic segment, until there are six stripes, of differing shape and length (figure 2*d*2–4)*.* Invagination begins as the final stripes are clearly defined and segmental *inv* expression is maintained throughout the germ-band stage (figure 2*d*5–6).

### (b) Mutual interactions of segmentation genes

#### (i) *even-skipped* knock-down

**Figure 3.**
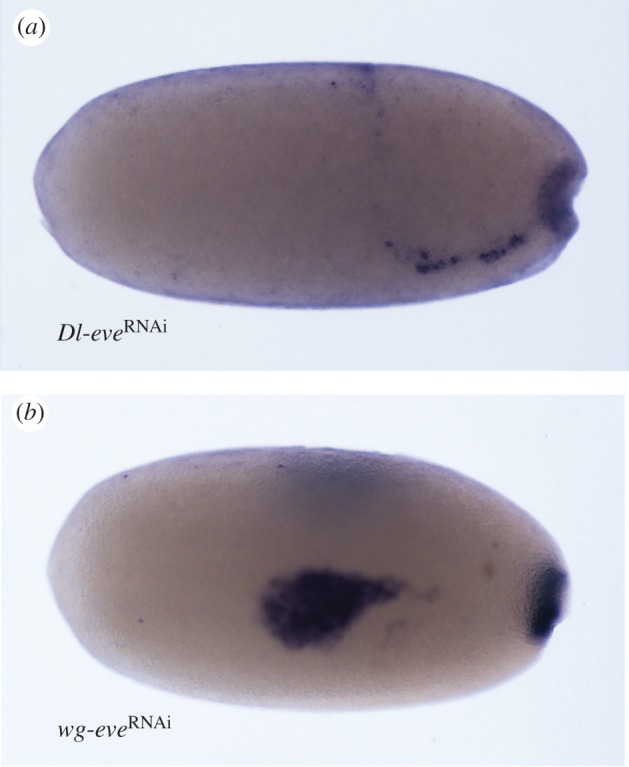
Expression of segmental genes following knock-down of *even-skipped*. (*a*) Early expression (32–34 hAEL) of *delta* is shifted posteriorly. (*b*) Expression of *wingless* at 34–36 hAEL is shifted anteriorly. The anterior pre-gnathal patch elongates relative to the normal expression (cf. figure 2*c*5–*c*6) and all gnathal and thoracic segmental stripes are lost.

The earliest gene to be expressed in a segmental manner is also the one that has the most severe effects when knocked down. Liu & Kaufman [24] showed that knocking down *eve* results in a complete loss of all gnathal, thoracic and abdominal segments, leaving only the pre-gnathal head intact. We wanted to see whether this effect is already seen at the level of the blastodermal segmental genes, or whether it is a result of a later role of *eve*. Knocking down *eve* through maternal RNAi affects the later expression pattern of *wg* and *Dl* (figure 3). The anterior early *Dl* stripe shifts to the posterior of the embryo and curves dorsally (figure 3*a*). The main six segmental stripes fail to form. In *wg*-stained embryos, we see the same shift and the deletion of the same segmental stripes while the anterior patch (ocular + antennal segments), which is elliptical in the wild-type, is larger and spreads to the posterior in *eve*^RNAi^ embryos (figure 3*b*). Surprisingly, the posterior expression patch that marks the growth zone in normal embryos is not disrupted, despite the loss of growth zone derived segments in *eve*^RNAi^ embryos.

These results are consistent with the idea raised by Liu & Kaufman [24] that it is the loss of the early broad domain of *eve* expression that leads to the severe RNAi phenotypes for this gene. Our results also establish *eve* as the highest of the four tested genes in the blastodermal segmentation cascade, consistent with its earlier expression.

#### (ii) *Delta* knock-down

The next gene in the temporal sequence of expression is *Dl.* Knocking down *Dl* through maternal RNAi did not affect the expression pattern of any of the other segmentation genes (*wg, eve, inv*) and invagination proceeded normally (electronic supplementary material, figure S1). However, pre-hatching larval phenotypes following RNAi (including larvae from the same clutches that showed normal blastodermal gene expression) exhibited severe posterior segmentation defects.

**Figure 4.**
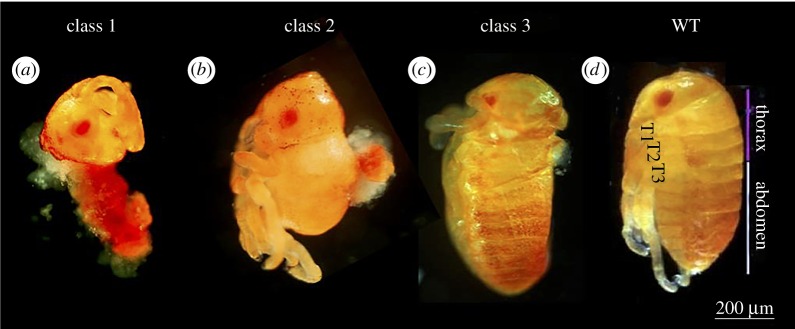
RNAi phenotypes of *delta* knock-down pre-hatching larvae, arranged according to severity. (*a*) Class 1 phenotypes: the anterior (pre-gnathal) head is nearly normal, but the gnathal head and trunk are fused and shrivelled. (*b*) Class 2 phenotypes: head and thorax are present and nearly normal, but the abdomen is truncated and various ectopic masses of cells are seen. (*c*) Class 3 phenotypes: all body regions are present but with anomalous morphologies. (*d*) Wild-type larva for comparison.

The most common and most severe phenotypes were defined as Class 1 (figure 4*a*). These larvae display a normal anterior head, but a fused and shapeless trunk. Class 2 larvae (figure 4*b*) show an apparently normal head and thorax with no abdomen, and shapeless masses of cells in different parts of the embryo. Class 3 (figure 4*c*) has a head, thorax and abdomen with a range of minor phenotypic defects. The distribution of the different classes is shown in table 1.

**Table 1.** Distribution of phenotype classes in *Dl* RNAi.

no. embryos	class 1	class 2	class 3	WT	no dev.
79	39	9	2	1	28

Given the normal expression of segmentation genes in the blastoderm, these phenotypes are surprising. Our interpretation is that Dl is not part of the segmentation cascade *per se*, and does not regulate *wg* or *inv* (and clearly not *eve,* which is upstream of it). However, it has a role in downstream tissue differentiation, so that its loss leads to a failure of development in all affected areas. Class 2 phenotypes as well as examination of *Dl*^RNAi^ germ-band stage embryos (electronic supplementary material, figure S2) suggest that *Dl*^RNAi^ disrupts posterior segmentation in the germ-band stage.

### (c) Regulation by gap genes

**Figure 5.**
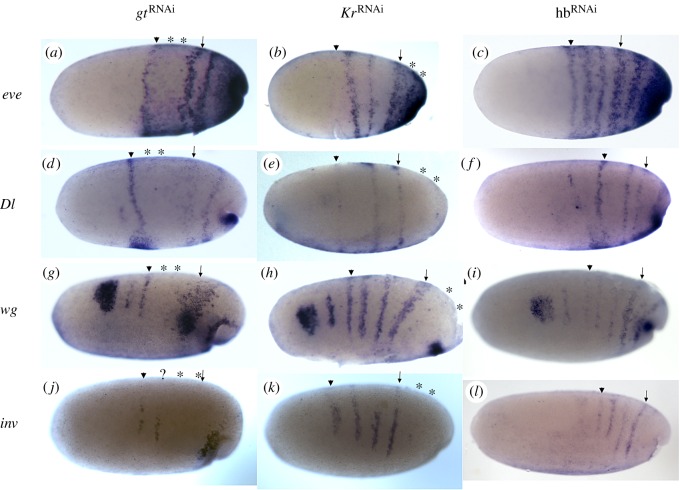
Expression of segmental genes following knock-down of gap genes. All embryos are at 34–36 hAEL, but note that the timing of these embryos is not as precise as those shown in figure 2*a–c*. Expression of *even-skipped,* (*d*–*f*) expression of *Delta*, (*g*–*i*) expression of *wingless* and (*j*–*l*) expression of *invected*, following RNAi against *giant* (left column), *Krüppel* (middle column) and *hunchback* (right column). In each embryo, the mandibular segment is marked with an arrowhead and the first thoracic segment with an arrow. Asterisks mark the putative location of missing segmental stripes. Knock-down of *giant* leads to the loss of the maxillary and labial segments and to disruption of the first thoracic segments. However, in the embryo imaged for *invected* (*j*) the labial and first thoracic segmental stripes are missing (this could be related to the position of the deleted stripes relative to the segmental/parasegmental borders). Knock-down of *Krüppel* leads to the loss of the second and third thoracic segments (occasionally also the first is missing—not shown). Knock-down of *hunchback* does not lead to the loss segmental expression in any of the genes at the blastoderm stage.

Since the expression pattern of the segment-polarity genes in the *Oncopeltus* blastoderm bears some similarities to that in *Drosophila,* we wanted to see whether the gap genes have an upstream role that is also similar to what is known from there. We knocked down the three gap genes *hunchback*, *Krüppel* and *giant* [23], and looked at the resulting blastodermal expression of the four segmentation genes discussed above (figure 5).

In *gt*^RNAi^ embryos, we find two segmental stripes, corresponding to the maxillary and labial segment, missing for all four genes (this was previously reported for *eve* [26]), as well as partial disruption of the stripe corresponding to the first thoracic segment (figure 5*a,d*,*g*,*j*)*.* These two segments are also the segments missing in *gt*^RNAi^ larvae [23,26]. In *Kr*^RNAi^ embryos, we see a loss of the stripes corresponding to thoracic segments 1/2–3 for all three genes (figure 5*b*,*e*,*h*,*k*). For *eve*, this is manifested as a fusion of the expression domains of the three thoracic segments (figure 5*b*), presumably as a result of the lack of clearing of the intersegmental regions for these segments. This is again consistent with the larval RNAi phenotype, which shows a loss of the same two–three segments [23,27]. In all cases, for both gap genes, the unaffected segmental stripes are expressed in their normal relative position, with no evidence for a shift. These results suggest a regulation of specific segments by these two gap genes. The gap genes may be regulating all four genes, but we believe it is more likely that they are directly regulating *eve*, and that Eve is then regulating the remaining genes. Based on what is known from *Drosophila*, it is possible that the regulation of *eve* is via segment-specific enhancer elements [28–31].

The situation for *hb* is different. According to Liu & Kaufman [19], *hb* is required for suppression of abdominal identity, and in *hb*^RNAi^ larvae, the maxillary to third thoracic segments take on abdominal fate. We see normal expression of all four blastodermal segmentation genes in *hb*^RNAi^ embryos (figure 5*c*,*f*,*i*,*l*), supporting the idea that *hb* has a role in defining segmental *identity*, but not in segment determination, and is thus very different in function from *gt* and *Kr*.

## 4. Discussion

The events of the late blastoderm patterning process in *Oncopeltus*, taking place between 32 and 36 hAEL, involve a nearly simultaneous definition of six segments at the molecular level. The sequence of appearance of segmental stripes for the four genes we looked at is *eve* → *Dl* → *wg* → *inv* (figure 2). The actual regulatory cascade is apparently similar, with *eve* being upstream of the three other genes, but *Dl* not involved in downstream regulation of segmental expression.

Above this sequence of segmentally expressed genes lies the gap-gene network [23,32]. At least two of the gene products acting at this earlier stage (Kr and Gt) regulate the formation of specific segmental expression stripes. A third (Hb) regulates segmental identity rather than the formation of specific segments.

Comparing this cascade to the *Drosophila* paradigmatic cascade reveals many similarities, but also intriguing differences. As in *Drosophila*, there is an early gap-gene stage, with individual genes controlling specific regions and segments. The expression of *eve* is similar in that it starts out uniform and gradually resolves to a periodic stripe pattern [29,33]. However, *eve* in *Drosophila* is expressed in alternating segments, whereas in *Oncopeltus* it is expressed in every segment. The expression of the segment-polarity genes *wg* and *inv/en* is the final stage in the segmentation cascade in both cases. The expression of *Dl* in segmental stripes is found in *Oncopeltus* only. The lack of a reference point and the lack of an obvious blastodermal phenotype make the role of *Dl* in blastoderm segmentation difficult to explain. We can speculate that the role of *Dl* in segmentation, in general, is a remnant of its role in generating a primary cycling pattern in ancestral sequential segmentation, but there are no current data to support such a speculation.

We describe the appearance of the segmental stripes of the four studied genes as ‘nearly simultaneous’. In all cases, the stripes come up over a period of about 2 h (out of a blastoderm stage of approx. 24 h), in a sequence that is clearly not anterior–posterior, in contrast to germ-band sequential segmentation. The sequence differs among the tested genes. For example, the first segmental expression of *eve* is in T1 while *inv* is first expressed in the mandibular segment. The supposedly simultaneous appearance of segment-polarity genes in *Drosophila* is in fact also only nearly simultaneous [34], and stripes come up over a period of approximately 10 minutes (out of a 2–3 h blastoderm stage).

In addition to the differences in precise timing, there are shape differences among the segmental stripes of different segments for each gene. For example, the mandibular stripe of *eve* is thinner than the rest of the *eve* segmental stripes, while the mandibular stripe of *Dl* is thicker than the rest of the *Dl* stripes. Similarly, *inv* and *wg* segmental stripes have different lengths and widths. The size and shape of a segmental stripe may depend of the size of the future segment, or the cell number of the future segment. The lack of correlation among the genes does not necessarily contradict this idea, as each gene may have a different role in the forming segment.

Given the large phylogenetic distance and the difference in general developmental pattern between *Drosophila* and *Oncopeltus*, the similarities in blastoderm segmentation are striking. Indeed, the two patterns are similar enough that it seems reasonable to conclude that they represent homologous processes. If this is true, it prompts a rethinking of what we know about the evolution of insect development, in general, and specifically of the evolution of segmentation modes in insects. Peel [35] hypothesized that the evolutionary transition between short and long germ development involved a gradual takeover of posterior segments by an anterior gap-gene-based simultaneous patterning system. The growth-zone-based sequential segmentation process gradually disappeared, and the anterior process patterned more and more segments, and eventually all segments.

**Figure 6.**
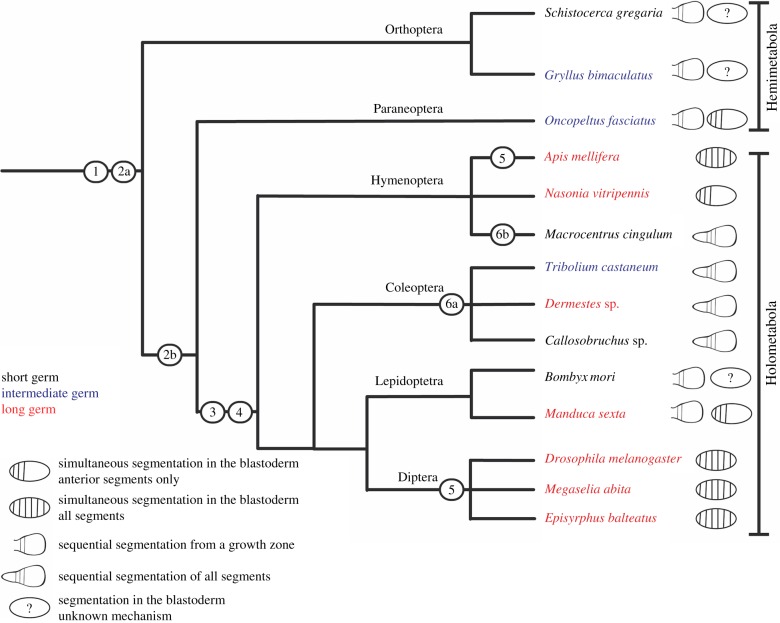
The evolution of segmentation modes in insects. This analysis looks not only at germ type but also at modes of segment generation (simultaneous versus sequential), and lists a series of hypothetical events that occurred through the evolution of the different modes. (1) Sequential segmentation is the plesiomorphic mode of segment generation in insects and is found in all non-insect arthropods. (2) The appearance of simultaneous segmentation in the blastoderm occurred either at the common ancestor of Holometabola + Paraneoptera (2b) or earlier in the insect lineage (2a). Not enough is known about blastodermal segmentation in Orthoptera, but from the little that is known, we suggest that simultaneous segmentation occurred after the splitting of Orthoptera (option 2b). (3) Heterochronic shifts lead to delayed gastrulation and gastrulation independent segmentation—long germ segmentation. Simultaneous segmentation expands to include more and more segments, but vestiges of a bi-phasic mode of segmentation remain. (4) Pair-rule patterning of segments also appeared at the base of Holometabola. (5) Sequential segmentation is lost completely in several lineages within Diptera and Hymenoptera, leaving extreme long germ simultaneous segmentation of all segments in the blastoderm stage. (6) Simultaneous segmentation is lost completely in some (or all) lineages within Coleoptera (6a), and in some parasitic wasps (6b), leaving sequential segmentation of all segments. Heterochronic shifts in different lineages lead to long, intermediate and short germ development, unrelated to the sequential mechanism of segment generation.

Building on this hypothesis and incorporating our results, we present a scenario for the evolution of long germ segmentation from the ancestral short germ segmentation mode (figure 6). This scenario does not distinguish merely between long and intermediate/short germ development, but looks at whether segments are generated sequentially or nearly simultaneously in different regions of the embryo. Sequential segmentation is unequivocally the ancestral mode for insects, and also probably for arthropods (figure 6 node 1). It is found in all hemimetabolous insects and in some branches of Holometabola. Simultaneous segmentation is found in three of the four branches of Holometabola for which we have data. This has usually been interpreted as suggesting repeated convergent evolution of simultaneous segmentation within Holometabola [15,36]. However, our results indicate that a very similar process, albeit patterning only some of the segments, is found in a close outgroup to Holometabola. This strongly suggests that the process of simultaneous segmentation is a plesiomorphic character for Holometabola, and evolved prior to the radiation of this clade (node 2), most likely at the base of Holometabola + Paraneoptera (node 2b). An acceleration of early development led to the delay of gastrulation until after the definition of the segments. This is the defining feature of long germ development in its original meaning, and we suggest it is an autapomorphy for Holometabola (node 3). The appearance of pair-rule patterning (i.e. the definition of segments in pairs, rather than individually) probably also occurred at the base of Holometabola (node 4). Vestiges of the ancestral bi-phasic segmentation mode can still be seen even in long germ species, such as *Nasonia vitripennis* where posterior segments are patterned sequentially [37]. Simultaneous segmentation ultimately expanded to pattern all of the segments, giving rise to the extreme long germ pattern seen in most Diptera and in many Hymenoptera (node 5). Conversely, simultaneous segmentation was lost completely at the base of Coleoptera (node 6), giving rise to the extreme sequential segmentation seen in *Tribolium*, wherein even the anterior-most blastoderm segments are patterned via a travelling wave of sequential patterning [38–40]. Intriguingly, within Coleoptera there are species that are defined as long, intermediate or short germ developers [5,41], but this reflects a difference in the relative timing of gastrulation relative to segmentation, and they all pattern segments sequentially. A similar loss occurred in some lineages of parasitic wasps.

The loss of sequential segmentation in extreme long germ embryos may have occurred once at the base of Holometabola or in several lineages in parallel. Several lines of evidence point to a single loss, followed by its re-evolution in, e.g. Coleoptera. Re-evolution of sequential segmentation within a simultaneously segmenting lineage has been conclusively demonstrated in parasitic wasps [42,43], indicating that such an evolutionary reversal is possible. In addition, sequential segmentation in non-insect arthropods is believed to be mediated by the Notch pathway [9–11,44]. Notch ligands have a demonstrated (if not entirely clear) role in segmentation in hemimetabolous insects [12–14], but not in holometabolous insects [35,45–47], including the sequentially segmenting beetle *Tribolium.* This is consistent with Notch-mediated sequential segmentation being lost at the base of Holometabola, and the re-evolved sequential segmentation being mediated by a different oscillator.

We suggest that simultaneous segmentation in a long germ embryo evolved at the base of Holometabola, using a pre-existing simultaneous segmentation mechanism in an intermediate germ ancestor (a situation still retained in *Oncopeltus*). The simultaneous segmentation was then modified, expanded or reduced in different lineages of holometabolous insects (figure 6). Given the phenomenal success of this group (holometabolous insects comprise more than half of all animal species), it is tempting to speculate that their success might be linked with the evolution of a novel development mode. While there is no convincing explanation for the linkage between simultaneous segmentation and the biphasic life history that characterizes the holometabolous insects, it has long been known that embryonic development is far more rapid in Holometabola compared with hemimetabolous insects [48]. Perhaps this acceleration of development was achieved through simultaneous segmentation, and this accelerated development facilitated the transition to a biphasic life history, which in turn paved the way for the subsequent adaptive radiation of holometabolous insects.

## Supplementary Material

Supplementary Figure 1

## Supplementary Material

Supplementary Figure 2
