# Supplementary figures and images for: Blastoderm segmentation in Oncopeltus fasciatus and the evolution of insect segmentation mechanisms
Source: Proc Biol Sci. 2016 Oct 12;283(1840):20161745. doi: 10.1098/rspb.2016.1745 (PMC5069518; doi:10.1098/rspb.2016.1745)

*Dl*-RNAi 36-38 hAEL

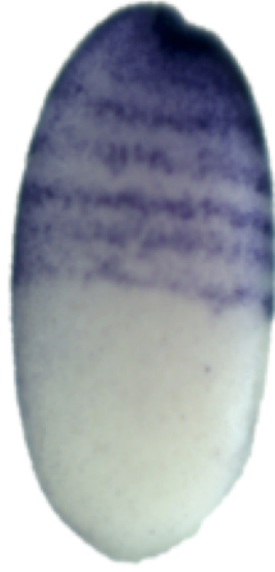

*eve*

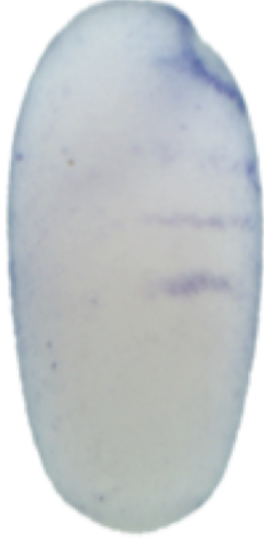

*inv*

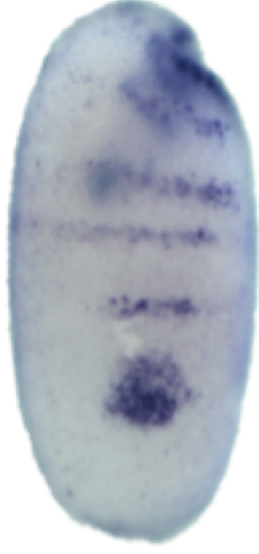

*wg*

Supplement: Supplementary Figure 1 [file rspb20161745supp1.pdf]

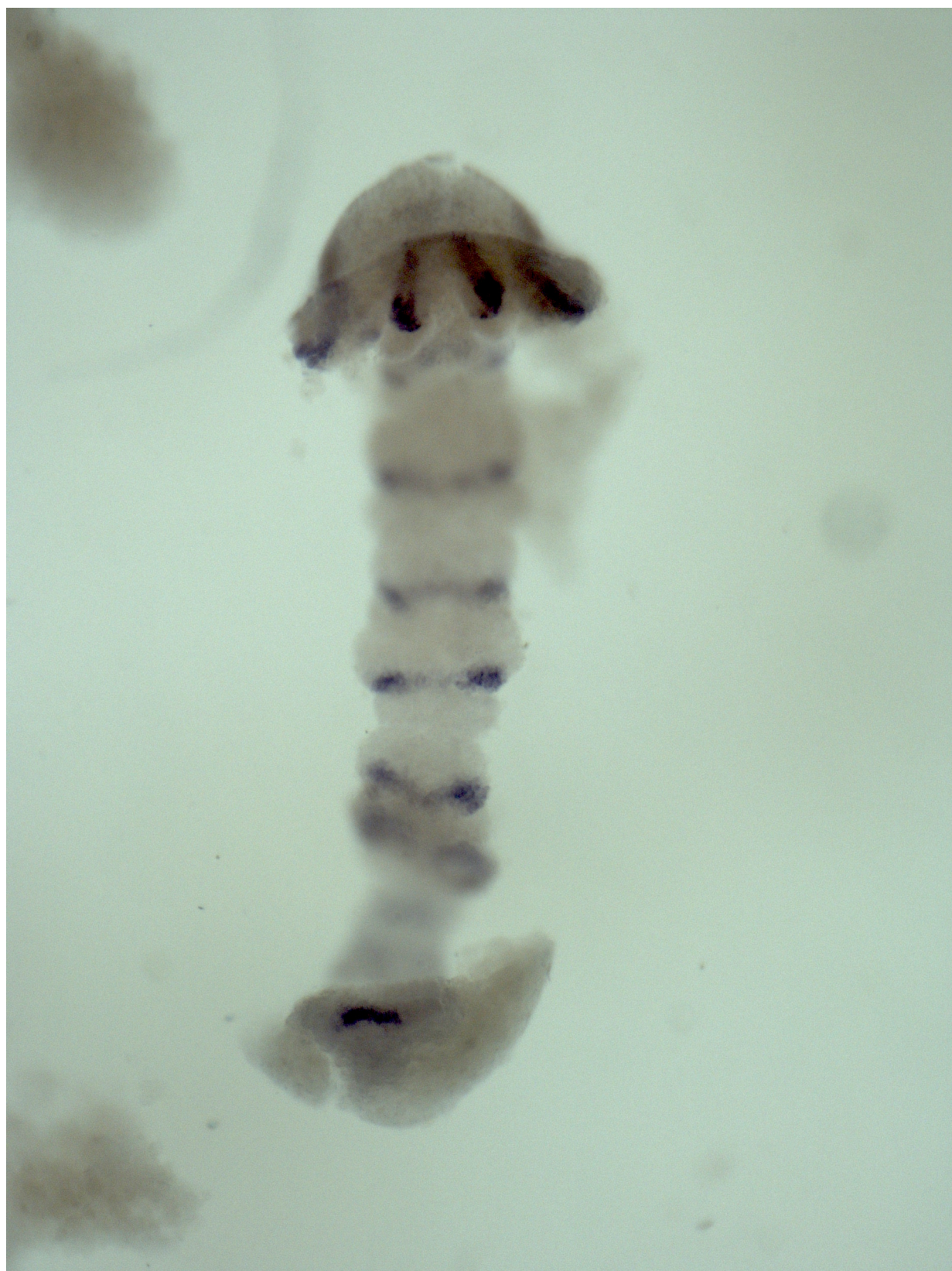

Supplement: Supplementary Figure 2 [file rspb20161745supp2.pdf]
